# 1,3-Diphenyl­propan-2-one (2,4-dinitro­phen­yl)hydrazone

**DOI:** 10.1107/S1600536810002746

**Published:** 2010-02-10

**Authors:** Ligia R. Gomes, Carlos F. R. A. C. Lima, Luís M. N. B. F. Santos, Paula Brandão, John Nicolson Low

**Affiliations:** a REQUIMTE, Departamento de Química e Bioquímica, Faculdade de Ciências, Universidade do Porto, Rua do Campo Alegre, 687, P-4169_007 Porto, Portugal; b Centro de Investigação em Química, Departamento de Química e Bioquímica, Faculdade de Ciências, Universidade do Porto, Rua do Campo Alegre, 687, P-4169_007 Porto, Portugal; c CICECO, Departamento de Química, Universidade de Aviero, 3810-193 Aveiro, Portugal; d Department of Chemistry, University of Aberdeen, Meston Walk, Old Aberdeen AB24 3UE, Scotland.

## Abstract

In the title compound, C_21_H_18_N_4_O_4_, there is an intra­molecular N—H⋯O hydrogen bond between the amino H atom and an O atom of the 2-nitro group of the adjacent benzene ring. The central benzene ring forms dihedral angles of 79.98 (7) and 82.88 (7)° with the two phenyl rings. In the crystal structure, mol­ecules are linked into a three-dimensional network by weak C—H⋯N, C—H⋯O and C—H⋯π inter­actions.

## Related literature

For the structures of related 2,4-dinitro­phenyl hydrazines, see: Wardell *et al.* (2006 ▶); Lima *et al.* (2009 ▶). For hydrogen-bond graph-set notation, see: Bernstein *et al.* (1995 ▶).
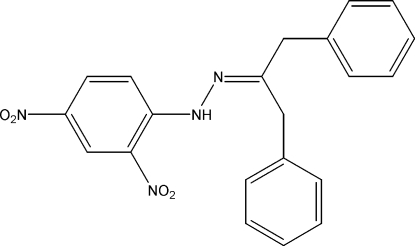


## Experimental

### 

#### Crystal data

C_21_H_18_N_4_O_4_
*M* *_r_* = 390.39
Monoclinic, 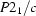

*a* = 17.2448 (9) Å
*b* = 5.1013 (2) Å
*c* = 22.7459 (13) Å
β = 109.475 (2)°
*V* = 1886.49 (16) Å^3^
*Z* = 4
Mo *K*α radiation
μ = 0.10 mm^−1^
*T* = 150 K
0.40 × 0.06 × 0.02 mm

#### Data collection

Bruker SMART APEXII diffractometer
Absorption correction: multi-scan (*SADABS*; Bruker, 2004 ▶) *T*_min_ = 0.962, *T*_max_ = 0.998
13123 measured reflections
4973 independent reflections
3417 reflections with *I* > 2σ(*I*)
*R*_int_ = 0.032

#### Refinement

*R*[*F* ^2^ > 2σ(*F* ^2^)] = 0.046
*wR*(*F* ^2^) = 0.126
*S* = 1.04
4973 reflections
262 parameters
H-atom parameters constrained
Δρ_max_ = 0.31 e Å^−3^
Δρ_min_ = −0.24 e Å^−3^

### 

Data collection: *APEX2* (Bruker, 2004 ▶); cell refinement: *APEX2* and *SAINT* (Bruker, 2004 ▶); data reduction: *SAINT*; program(s) used to solve structure: *SHELXS97* (Sheldrick, 2008 ▶); program(s) used to refine structure: *SHELXL97* (Sheldrick, 2008 ▶); molecular graphics: *ORTEPII* (Johnson, 1976 ▶) and *PLATON* (Spek, 2009 ▶); software used to prepare material for publication: *SHELXL97*.

## Supplementary Material

Crystal structure: contains datablocks global, I. DOI: 10.1107/S1600536810002746/lh2985sup1.cif

Structure factors: contains datablocks I. DOI: 10.1107/S1600536810002746/lh2985Isup2.hkl

Additional supplementary materials:  crystallographic information; 3D view; checkCIF report

## Figures and Tables

**Table 1:** Hydrogen-bond geometry (Å, °) *Cg*31 and *Cg*41 are the centroids of the C31–C36 and C41–C46 phenyl rings, respectively.

*D*—H⋯*A*	*D*—H	H⋯*A*	*D*⋯*A*	*D*—H⋯*A*
N1—H1⋯O122	0.91	1.92	2.5976 (15)	129
C15—H15⋯O142^i^	0.95	2.44	3.314 (2)	153
C3—H3*A*⋯N2^ii^	0.99	2.53	3.3811 (18)	144
C3—H3*B*⋯O121^iii^	0.99	2.55	3.3138 (18)	134
C4—H4*B*⋯*Cg*41^iv^	0.99	2.79	3.7438 (16)	163
C45—H45⋯*Cg*31^v^	0.95	2.92	3.7424 (18)	145

Symmetry codes: (i) 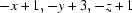; (ii) 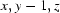; (iii) 
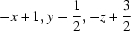
; (iv) 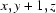; (v) 
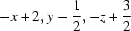
.

**Fig. 1.**
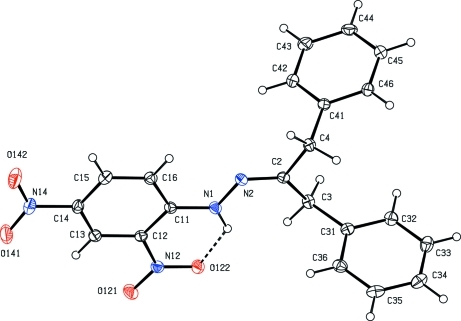
A view of (1) with our numbering scheme. Displacement ellipsoids are drawn at the 30% probability level.

**Fig. 2.**
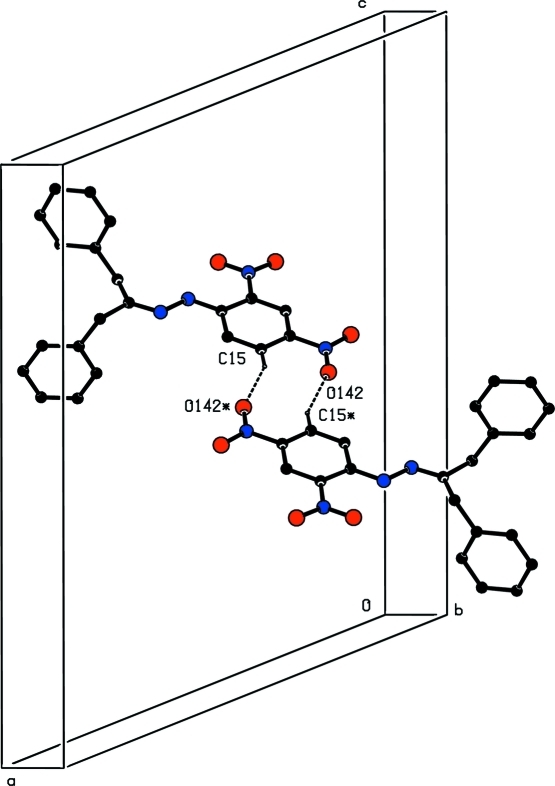
A view of the R22(10) dimer. The atoms labelled * are in the molecule at (1-x,3-y, 1-z). Hydrogen atoms not involved in the motifs are not included.

**Fig. 3.**
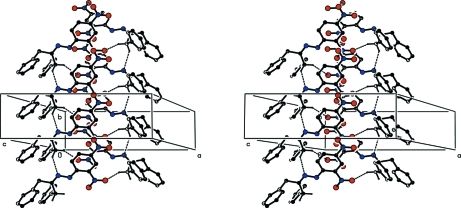
A stereoview of part of the crystal structure of compound, showing part of the tubular structure running parallel to the b-axis formed by C—H..O and C—H..N hydrogen bonds. Hydrogen atoms not involved in the motifs are not included nor are the reinforcing C—H···π interaction which is omitted for the sake of clarity.

## supplementary crystallographic information

### Comment

The molecular structure of the title compound with the crystallographic numbering scheme is shown in Figure. 1. The relevant bonds, angles and distances compare well with similar structures (Lima *et al.*, 2009; Wardell *et al.*,  2006).

The molecular geometry and conformation is as expected taking account of electronic repulsions and steric effects.

The orange colour of the title compound is caused by the conjugation of the nitrophenyl with the -N-N= group. The atoms N1, N2 C2, C3 and C4 are coplanar with a rms deviation of the fitted atoms of 0.0126 Å. The two phenyl groups attached to C3 and C4 lie out of this plane. The dihedral angle between the mean plane of the the C11-C16 benzene ring and C31-C36 phenyl ring is 79.98 (7)° and that between the C11-C16 ring C41-46 ring is 82.88 (7)°. The dihedral angle formed by the mean planes of the C31-C36 and C41-C46 phenyl rings 16.25 (8)°.

An intramolecular hydrogen bond, N1-H1···O122, forms an R(6) ring, Bernstein *et al.*, (1995) as in (E)-1-phenylbutan-2-one(2,4-dinitrophenyl)hydrazone (Lima *et al.*, 2009). In addition to this hydrogen bond there are three weak intermolecular hydrogen bonds and two C-H···π interactions which link the molecules into a three-dimensional network.

C15 via H15(x,y,z) forms a hydrogen bond to O142(1-x, 3-y, 1-z), forming an R^2^_2_(10) ring thus creating a centrosymmetric dimer centred on the crystallographic centre-of-symmetry at (0.5, 1.5, 0.5), Figure 2. The other two hydrogen bonds involve the hydrogen atoms attached to C3, these two hydrogen bonds along with a C–H..π interaction form a tubular structure which runs parallel to the b-axis, Figure 3. C3 via H3A(x,y,z) forms a hydrogen bond to N2(x,-1+y,z) forming a C4 chain parallel to the b axis. C3 via H3B(x,y,z) forms a hydrogen bond to O121(1-x,-1/2+y,3/2-z) forming a C9 helical chain produced by the action of a screw axis at (0.5,y,0.75) which also runs parallel to the b-axis. The resulting tubular structure is further reinforced by a weak C-H..π interaction, C4—H4B···Cg41(x, y+1, z) where Cg41 is the centre of gravity of the phenyl ring containing C41. The b-axis tubular structures are connected by the R^2^_2_(10) rings and by a second weak C-H..π interaction, C45—H45···Cg31(-x+2, y-1/2, -z+3/2) where Cg31 is the centre of gravity of the phenyl ring containing C31, to form a three dimensional network. A short nitro-nitro contact of 2.8506 (15)Å between N12(x,y,z) and O122(1-x,-1/2+y,3/2-z) is observed. A similar short contact of 2.755 (2)Å occurs in (E)-1-phenylbutan-2-one(2,4-dinitrophenyl)hydrazone (Lima *et al.*,2009).

### Experimental

(1) was obtained from the condensation reaction of dibenzylketone with 2,4-dinitrophenylhydrazine. 2.3 mmol of dibenzylketone was added to a solution of 2.4 mmol of 2,4-dinitrophenylhydrazine in an ethanol /HCl mixture (10:1 and heated (50 °C) to reflux until completely dissolved. The reaction mixture was extracted with ethylacetate and then removed under vacuum. The resulting orange residue was re-crystallised, first from ethanol and then from ethylacetate. (overall yield: 0.54 g, 60%). 1*H*-NMR (400 MHz, CDCl_3_, 298 K, TMS): δ = 11.22 (s, 1H, H_5_), δ = 9.11 (d, J = 2.8 Hz, 1H, H_8_), δ = 8.34 (dd, J = 9.6 Hz, J = 2.8 Hz, 1H, H_7_), δ = 8.04 (d, J = 9.6 Hz, 1H, H_6_), δ = [7.40-7.25] (m, 8H, H_1_ - H_3_), δ = 7.18 (d, J = 6.8 Hz, 2H, H_3_), δ = 3.83 (s, 2H, H_4_), δ = 3.75 (s, 2H, H_4_).

Orange needles suitable for X-ray diffraction were grown from dichloromethane.

### Refinement

Molecule (1) crystallized in the monoclinic system; space group P2_1_/c. H atoms were treated as riding atoms with C—H(aromatic), 0.95 Å, C—H(CH_2_), 0.99 Å. The atom attached to N1 was located on a difference map at a distance of 0.9123Å and was fixed as a riding atom at this distance.

### Crystal data

**Table:**

C_21_H_18_N_4_O_4_	*D*_x_ = 1.375 Mg m^−^^3^
*M**_r_* = 390.39	Melting point: 381.7 K
Monoclinic, *P*2_1_/*c*	Mo *K*α radiation, λ = 0.71073 Å
*a* = 17.2448 (9) Å	Cell parameters from 211 reflections
*b* = 5.1013 (2) Å	θ = 15.5–51.4°
*c* = 22.7459 (13) Å	µ = 0.10 mm^−^^1^
β = 109.475 (2)°	*T* = 150 K
*V* = 1886.49 (16) Å^3^	Needle, orange
*Z* = 4	0.40 × 0.06 × 0.02 mm
*F*(000) = 816	

### Data collection

**Table:**

Bruker SMART APEX diffractometer	4973 independent reflections
Radiation source: fine-focus sealed tube	3417 reflections with *I* > 2σ(*I*)
graphite	*R*_int_ = 0.032
ω scans	θ_max_ = 29.1°, θ_min_ = 2.6°
Absorption correction: multi-scan (*SADABS*; Bruker, 2004)	*h* = −22→23
*T*_min_ = 0.962, *T*_max_ = 0.998	*k* = −5→6
13123 measured reflections	*l* = −29→31

### Refinement

**Table:**

Refinement on *F*^2^	Primary atom site location: structure-invariant direct methods
Least-squares matrix: full	Secondary atom site location: difference Fourier map
*R*[*F*^2^ > 2σ(*F*^2^)] = 0.046	Hydrogen site location: inferred from neighbouring sites
*wR*(*F*^2^) = 0.126	H-atom parameters constrained
*S* = 1.04	*w* = 1/[σ^2^(*F*_o_^2^) + (0.0644*P*)^2^ + 0.0648*P*] where *P* = (*F*_o_^2^ + 2*F*_c_^2^)/3
4973 reflections	(Δ/σ)_max_ < 0.001
262 parameters	Δρ_max_ = 0.31 e Å^−^^3^
0 restraints	Δρ_min_ = −0.24 e Å^−^^3^

### Special details

**Table:**

Geometry. All esds (except the esd in the dihedral angle between two l.s. planes) are estimated using the full covariance matrix. The cell esds are taken into account individually in the estimation of esds in distances, angles and torsion angles; correlations between esds in cell parameters are only used when they are defined by crystal symmetry. An apprO122imate (isotropic) treatment of cell esds is used for estimating esds involving l.s. planes.
Refinement. Refinement of *F*^2^ against ALL reflections. The weighted *R*-factor wR and goodness of fit *S* are based on *F*^2^, conventional *R*-factors *R* are based on *F*, with *F* set to zero for negative *F*^2^. The threshold expression of *F*^2^ > σ(*F*^2^) is used only for calculating *R*-factors(gt) etc. and is not relevant to the choice of reflections for refinement. *R*-factors based on *F*^2^ are statistically about twice as large as those based on *F*, and *R*- factors based on ALL data will be even larger.

### Fractional atomic coordinates and isotropic or equivalent isotropic displacement parameters (Å^2^)

**Table:**

	*x*	*y*	*z*	*U*_iso_*/*U*_eq_	
O121	0.38029 (6)	0.5908 (2)	0.68188 (5)	0.0370 (3)	
O122	0.50403 (6)	0.43658 (19)	0.71259 (5)	0.0290 (2)	
O141	0.29905 (7)	1.3039 (3)	0.54043 (6)	0.0478 (3)	
O142	0.38465 (7)	1.4722 (2)	0.49984 (6)	0.0488 (3)	
N1	0.61933 (7)	0.6614 (2)	0.68083 (5)	0.0238 (3)	
H1	0.6085	0.5463	0.7078	0.029*	
N2	0.69791 (6)	0.7018 (2)	0.67903 (5)	0.0223 (3)	
N12	0.45159 (7)	0.5957 (2)	0.68209 (5)	0.0249 (3)	
N14	0.36726 (8)	1.3166 (3)	0.53486 (6)	0.0328 (3)	
C2	0.75430 (8)	0.5395 (3)	0.70889 (6)	0.0210 (3)	
C3	0.74525 (8)	0.3003 (3)	0.74514 (6)	0.0245 (3)	
H3A	0.7560	0.1417	0.7240	0.029*	
H3B	0.6878	0.2904	0.7450	0.029*	
C4	0.83751 (8)	0.5887 (3)	0.70326 (6)	0.0235 (3)	
H4A	0.8796	0.5883	0.7454	0.028*	
H4B	0.8379	0.7640	0.6847	0.028*	
C11	0.55764 (8)	0.8161 (3)	0.64553 (6)	0.0218 (3)	
C12	0.47530 (8)	0.7921 (3)	0.64507 (6)	0.0219 (3)	
C13	0.41292 (8)	0.9552 (3)	0.60887 (6)	0.0250 (3)	
H13	0.3583	0.9382	0.6095	0.030*	
C14	0.43201 (8)	1.1413 (3)	0.57223 (6)	0.0261 (3)	
C15	0.51178 (9)	1.1705 (3)	0.57056 (6)	0.0277 (3)	
H15	0.5233	1.3002	0.5446	0.033*	
C16	0.57319 (8)	1.0114 (3)	0.60642 (6)	0.0255 (3)	
H16	0.6274	1.0320	0.6051	0.031*	
C31	0.80301 (8)	0.3014 (3)	0.81217 (6)	0.0231 (3)	
C32	0.86423 (9)	0.1136 (3)	0.83308 (7)	0.0275 (3)	
H32	0.8710	−0.0142	0.8048	0.033*	
C33	0.91584 (9)	0.1099 (3)	0.89490 (7)	0.0331 (4)	
H33	0.9575	−0.0199	0.9087	0.040*	
C34	0.90638 (10)	0.2952 (3)	0.93612 (7)	0.0358 (4)	
H34	0.9411	0.2920	0.9785	0.043*	
C35	0.84621 (10)	0.4858 (3)	0.91564 (7)	0.0351 (4)	
H35	0.8402	0.6149	0.9439	0.042*	
C36	0.79469 (9)	0.4891 (3)	0.85398 (7)	0.0287 (3)	
H36	0.7535	0.6203	0.8403	0.034*	
C41	0.85952 (8)	0.3836 (3)	0.66334 (6)	0.0223 (3)	
C42	0.80419 (9)	0.3234 (3)	0.60454 (6)	0.0338 (4)	
H42	0.7530	0.4126	0.5897	0.041*	
C43	0.82283 (10)	0.1359 (4)	0.56759 (7)	0.0397 (4)	
H43	0.7843	0.0962	0.5277	0.048*	
C44	0.89716 (10)	0.0059 (3)	0.58834 (7)	0.0327 (4)	
H44	0.9097	−0.1244	0.5630	0.039*	
C45	0.95344 (9)	0.0661 (3)	0.64620 (7)	0.0298 (3)	
H45	1.0051	−0.0206	0.6604	0.036*	
C46	0.93414 (8)	0.2534 (3)	0.68334 (6)	0.0256 (3)	
H46	0.9728	0.2928	0.7232	0.031*	

### Atomic displacement parameters (Å^2^)

**Table:**

	*U*^11^	*U*^22^	*U*^33^	*U*^12^	*U*^13^	*U*^23^
O121	0.0247 (5)	0.0451 (7)	0.0453 (6)	0.0008 (5)	0.0172 (5)	0.0075 (5)
O122	0.0301 (5)	0.0275 (5)	0.0303 (5)	0.0038 (4)	0.0111 (4)	0.0049 (4)
O141	0.0297 (6)	0.0569 (8)	0.0527 (7)	0.0135 (6)	0.0083 (5)	0.0170 (6)
O142	0.0399 (6)	0.0496 (8)	0.0480 (7)	0.0041 (6)	0.0029 (6)	0.0241 (6)
N1	0.0217 (6)	0.0243 (6)	0.0266 (6)	0.0007 (5)	0.0099 (5)	0.0023 (5)
N2	0.0203 (5)	0.0222 (6)	0.0254 (6)	−0.0006 (5)	0.0092 (4)	−0.0016 (4)
N12	0.0249 (6)	0.0266 (6)	0.0240 (6)	0.0001 (5)	0.0091 (5)	−0.0027 (5)
N14	0.0307 (7)	0.0329 (7)	0.0275 (6)	0.0025 (6)	−0.0001 (5)	0.0023 (5)
C2	0.0228 (6)	0.0196 (7)	0.0211 (6)	−0.0016 (5)	0.0080 (5)	−0.0041 (5)
C3	0.0214 (6)	0.0212 (7)	0.0302 (7)	−0.0019 (6)	0.0076 (5)	0.0000 (5)
C4	0.0204 (6)	0.0234 (7)	0.0266 (7)	−0.0016 (6)	0.0076 (5)	−0.0002 (5)
C11	0.0230 (6)	0.0209 (7)	0.0212 (6)	0.0017 (6)	0.0068 (5)	−0.0048 (5)
C12	0.0248 (6)	0.0215 (7)	0.0200 (6)	−0.0008 (6)	0.0083 (5)	−0.0022 (5)
C13	0.0232 (6)	0.0282 (7)	0.0221 (6)	0.0003 (6)	0.0057 (5)	−0.0059 (5)
C14	0.0263 (7)	0.0259 (7)	0.0216 (6)	0.0026 (6)	0.0021 (5)	−0.0021 (5)
C15	0.0328 (7)	0.0258 (8)	0.0229 (7)	−0.0020 (6)	0.0072 (6)	0.0011 (5)
C16	0.0241 (7)	0.0277 (7)	0.0253 (7)	−0.0015 (6)	0.0088 (6)	−0.0003 (5)
C31	0.0246 (7)	0.0210 (7)	0.0258 (7)	−0.0050 (6)	0.0109 (5)	0.0023 (5)
C32	0.0305 (7)	0.0220 (7)	0.0305 (7)	−0.0003 (6)	0.0107 (6)	−0.0003 (6)
C33	0.0322 (8)	0.0298 (8)	0.0337 (8)	0.0000 (7)	0.0065 (6)	0.0075 (6)
C34	0.0398 (9)	0.0411 (9)	0.0249 (7)	−0.0088 (8)	0.0088 (6)	0.0040 (6)
C35	0.0467 (9)	0.0353 (9)	0.0288 (8)	−0.0082 (8)	0.0197 (7)	−0.0066 (6)
C36	0.0315 (7)	0.0257 (7)	0.0332 (8)	−0.0003 (6)	0.0164 (6)	0.0009 (6)
C41	0.0211 (6)	0.0239 (7)	0.0232 (6)	−0.0011 (6)	0.0092 (5)	0.0023 (5)
C42	0.0255 (7)	0.0503 (10)	0.0230 (7)	0.0079 (7)	0.0046 (6)	−0.0003 (6)
C43	0.0326 (8)	0.0615 (11)	0.0219 (7)	0.0024 (8)	0.0050 (6)	−0.0098 (7)
C44	0.0375 (8)	0.0389 (9)	0.0263 (7)	0.0010 (7)	0.0166 (6)	−0.0063 (6)
C45	0.0270 (7)	0.0340 (8)	0.0290 (7)	0.0057 (6)	0.0102 (6)	0.0022 (6)
C46	0.0217 (6)	0.0302 (8)	0.0226 (6)	−0.0005 (6)	0.0043 (5)	−0.0011 (6)

### Geometric parameters (Å, °)

**Table:**

O121—N12	1.2281 (15)	C15—H15	0.9500
O122—N12	1.2409 (15)	C16—H16	0.9500
O141—N14	1.2261 (16)	C31—C32	1.387 (2)
O142—N14	1.2305 (17)	C31—C36	1.390 (2)
N1—C11	1.3528 (17)	C32—C33	1.3911 (19)
N1—N2	1.3847 (15)	C32—H32	0.9500
N1—H1	0.9123	C33—C34	1.379 (2)
N2—C2	1.2854 (17)	C33—H33	0.9500
N12—C12	1.4525 (18)	C34—C35	1.385 (2)
N14—C14	1.4615 (17)	C34—H34	0.9500
C2—C4	1.5036 (19)	C35—C36	1.388 (2)
C2—C3	1.5093 (19)	C35—H35	0.9500
C3—C31	1.5180 (18)	C36—H36	0.9500
C3—H3A	0.9900	C41—C46	1.3835 (18)
C3—H3B	0.9900	C41—C42	1.3939 (18)
C4—C41	1.5140 (19)	C42—C43	1.380 (2)
C4—H4A	0.9900	C42—H42	0.9500
C4—H4B	0.9900	C43—C44	1.379 (2)
C11—C16	1.420 (2)	C43—H43	0.9500
C11—C12	1.4218 (18)	C44—C45	1.3843 (19)
C12—C13	1.3917 (18)	C44—H44	0.9500
C13—C14	1.374 (2)	C45—C46	1.388 (2)
C13—H13	0.9500	C45—H45	0.9500
C14—C15	1.397 (2)	C46—H46	0.9500
C15—C16	1.3672 (19)		
			
C11—N1—N2	118.64 (11)	C15—C16—C11	121.32 (13)
C11—N1—H1	118.5	C15—C16—H16	119.3
N2—N1—H1	122.5	C11—C16—H16	119.3
C2—N2—N1	117.64 (11)	C32—C31—C36	118.78 (13)
O121—N12—O122	122.07 (12)	C32—C31—C3	120.76 (12)
O121—N12—C12	119.13 (11)	C36—C31—C3	120.46 (12)
O122—N12—C12	118.80 (11)	C31—C32—C33	120.88 (14)
O141—N14—O142	123.39 (12)	C31—C32—H32	119.6
O141—N14—C14	118.74 (13)	C33—C32—H32	119.6
O142—N14—C14	117.87 (13)	C34—C33—C32	119.82 (14)
N2—C2—C4	115.01 (12)	C34—C33—H33	120.1
N2—C2—C3	127.69 (13)	C32—C33—H33	120.1
C4—C2—C3	117.19 (11)	C33—C34—C35	119.86 (14)
C2—C3—C31	113.14 (11)	C33—C34—H34	120.1
C2—C3—H3A	109.0	C35—C34—H34	120.1
C31—C3—H3A	109.0	C34—C35—C36	120.26 (14)
C2—C3—H3B	109.0	C34—C35—H35	119.9
C31—C3—H3B	109.0	C36—C35—H35	119.9
H3A—C3—H3B	107.8	C35—C36—C31	120.39 (14)
C2—C4—C41	111.89 (11)	C35—C36—H36	119.8
C2—C4—H4A	109.2	C31—C36—H36	119.8
C41—C4—H4A	109.2	C46—C41—C42	118.28 (13)
C2—C4—H4B	109.2	C46—C41—C4	121.75 (11)
C41—C4—H4B	109.2	C42—C41—C4	119.96 (12)
H4A—C4—H4B	107.9	C43—C42—C41	120.87 (13)
N1—C11—C16	120.40 (12)	C43—C42—H42	119.6
N1—C11—C12	122.68 (12)	C41—C42—H42	119.6
C16—C11—C12	116.92 (12)	C44—C43—C42	120.23 (13)
C13—C12—C11	121.72 (13)	C44—C43—H43	119.9
C13—C12—N12	116.36 (12)	C42—C43—H43	119.9
C11—C12—N12	121.92 (11)	C43—C44—C45	119.72 (14)
C14—C13—C12	118.55 (13)	C43—C44—H44	120.1
C14—C13—H13	120.7	C45—C44—H44	120.1
C12—C13—H13	120.7	C44—C45—C46	119.81 (13)
C13—C14—C15	121.84 (12)	C44—C45—H45	120.1
C13—C14—N14	118.98 (13)	C46—C45—H45	120.1
C15—C14—N14	119.16 (13)	C41—C46—C45	121.07 (12)
C16—C15—C14	119.63 (13)	C41—C46—H46	119.5
C16—C15—H15	120.2	C45—C46—H46	119.5
C14—C15—H15	120.2		
			
C11—N1—N2—C2	−174.48 (12)	C13—C14—C15—C16	−0.3 (2)
N1—N2—C2—C4	177.96 (11)	N14—C14—C15—C16	178.54 (12)
N1—N2—C2—C3	1.8 (2)	C14—C15—C16—C11	0.0 (2)
N2—C2—C3—C31	−124.82 (14)	N1—C11—C16—C15	−179.75 (13)
C4—C2—C3—C31	59.11 (16)	C12—C11—C16—C15	0.70 (19)
N2—C2—C4—C41	−108.55 (13)	C2—C3—C31—C32	−115.42 (15)
C3—C2—C4—C41	68.02 (15)	C2—C3—C31—C36	65.49 (17)
N2—N1—C11—C16	1.50 (18)	C36—C31—C32—C33	0.8 (2)
N2—N1—C11—C12	−178.97 (11)	C3—C31—C32—C33	−178.27 (13)
N1—C11—C12—C13	179.34 (12)	C31—C32—C33—C34	−0.1 (2)
C16—C11—C12—C13	−1.12 (19)	C32—C33—C34—C35	−0.8 (2)
N1—C11—C12—N12	−0.91 (19)	C33—C34—C35—C36	0.9 (2)
C16—C11—C12—N12	178.63 (12)	C34—C35—C36—C31	−0.1 (2)
O121—N12—C12—C13	−3.20 (18)	C32—C31—C36—C35	−0.7 (2)
O122—N12—C12—C13	176.22 (12)	C3—C31—C36—C35	178.37 (13)
O121—N12—C12—C11	177.04 (12)	C2—C4—C41—C46	−129.80 (14)
O122—N12—C12—C11	−3.54 (18)	C2—C4—C41—C42	50.75 (18)
C11—C12—C13—C14	0.8 (2)	C46—C41—C42—C43	1.0 (2)
N12—C12—C13—C14	−178.94 (11)	C4—C41—C42—C43	−179.56 (15)
C12—C13—C14—C15	−0.1 (2)	C41—C42—C43—C44	−0.4 (3)
C12—C13—C14—N14	−178.95 (12)	C42—C43—C44—C45	−0.6 (3)
O141—N14—C14—C13	4.8 (2)	C43—C44—C45—C46	1.1 (2)
O142—N14—C14—C13	−176.13 (13)	C42—C41—C46—C45	−0.5 (2)
O141—N14—C14—C15	−174.14 (13)	C4—C41—C46—C45	−179.95 (13)
O142—N14—C14—C15	4.95 (19)	C44—C45—C46—C41	−0.5 (2)

### Hydrogen-bond geometry (Å, °)

**Table:**

Cg31 and Cg41 are the centroids of the C31–C36 and C41–C46 phenyl rings, respectively.

**Table:**

*D*—H···*A*	*D*—H	H···*A*	*D*···*A*	*D*—H···*A*
N1—H1···O122	0.91	1.92	2.5976 (15)	129
C15—H15···O142^i^	0.95	2.44	3.314 (2)	153
C3—H3A···N2^ii^	0.99	2.53	3.3811 (18)	144
C3—H3B···O121^iii^	0.99	2.55	3.3138 (18)	134
C4—H4B···Cg41^iv^	0.99	2.79	3.7438 (16)	163
C45—H45···Cg31^v^	0.95	2.92	3.7424 (18)	145

Symmetry codes: (i) −*x*+1, −*y*+3, −*z*+1; (ii) *x*, *y*−1, *z*; (iii) −*x*+1, *y*−1/2, −*z*+3/2; (iv) *x*, *y*+1, *z*; (v) −*x*+2, *y*−1/2, −*z*+3/2.

## References

[bb1] Bernstein, J., Davis, R. E., Shimoni, I. & Chang, N.-L. (1995). *Angew. Chem. Int. Ed. Engl.* **34**, 1555–1573.

[bb2] Bruker (2004). *APEX2*, *SAINT* and *SADABS* Bruker AXS Inc., Madison, Wisconsin, USA.

[bb3] Johnson, C. K. (1976). *ORTEPII* Report ORNL-5138. Oak Ridge National Laboratory, Tennessee, USA.

[bb4] Lima, C. F. R. A. C., Gomes, L. R., Santos, L. M. N. B. F., Rodriguez-Borges, J. E. & Low, J. N. (2009). *Acta Cryst.* E**65**, o2729. 10.1107/S1600536809041178 PMC2971069 21578325

[bb5] Sheldrick, G. M. (2008). *Acta Cryst.* A**64**, 112–122. 10.1107/S0108767307043930 18156677

[bb6] Spek, A. L. (2009). *Acta Cryst.* D**65**, 148–155. 10.1107/S090744490804362X PMC2631630 19171970

[bb7] Wardell, J. L., Low, J. N. & Glidewell, C. (2006). *Acta Cryst.* C**62**, o318–o320. 10.1107/S0108270106013114 16763315

